# Impact of Hypertrophic Cardiomyopathy on In-hospital Outcomes in Acute Ischemic Stroke

**DOI:** 10.19102/icrm.2026.17105

**Published:** 2026-10-15

**Authors:** Akhilesh Babbili, Sahith Reddy Thotamgari, Rahul Vyas, Prabandh Reddy Buchhanolla, Vijaywant Brar

**Affiliations:** 1 Department of Internal Medicine, Louisiana State University Health Sciences Center, Shreveport, LA, USA; 2 Division of Cardiology, Louisiana State University Health Sciences Center, Shreveport, LA, USA; 3 Department of Neurology, Louisiana State University Health Sciences Center, Shreveport, LA, USA

**Keywords:** Acute ischemic stroke, hypertrophic cardiomyopathy, mortality, National Inpatient Sample

## Abstract

The objective of this study was to assess the impact of hypertrophic cardiomyopathy (HCM) in hospitalizations with acute ischemic stroke (AIS). We used the National Inpatient Sample database between January 2016 and December 2019 to identify hospitalizations with an index diagnosis of AIS, using the International Classification of Diseases, Tenth Revision (ICD-10) codes. The cohort was further classified into two groups: patients with and without HCM. Propensity score matching (PSM) was used to balance covariates between the HCM and non-HCM groups. Multivariate logistic regression analysis was used to evaluate the association of outcomes, and all statistical analyses were conducted with weighted values. Our study encompassed 1,566,915 hospitalizations with a primary diagnosis of AIS, of which 3895 (0.25%) had a coexisting diagnosis of HCM. After 1:4 PSM, 3895 hospitalizations were included in the HCM group, and 15,580 hospitalizations were included in the control group. Logistic regression analysis revealed that HCM was associated with higher all-cause in-hospital mortality (odds ratio, 1.8; 95% confidence interval, 1.5–2.1; *P* < .001), longer hospital stays, and higher hospitalization costs compared to those without HCM. In the HCM group, age, congestive heart failure, peripheral vascular disease, renal failure, and coagulopathy were identified as predictors of mortality. In conclusion, we found that patients hospitalized with AIS and concurrent HCM were associated with worse in-hospital outcomes, including higher mortality, longer hospital stays, and increased hospitalization costs.

**CLINICAL IMPACT:**

Ischemic stroke is a known complication of hypertrophic cardiomyopathy (HCM), yet we know surprisingly little about how these patients do once they are admitted with an acute stroke. Using a large national inpatient database and a propensity-matched comparison, we found that stroke patients with HCM had nearly twice the odds of dying in the hospital, stayed longer, and had higher hospitalization costs than comparable patients without HCM. One finding we did not expect was that atrial fibrillation, often seen as the main driver of stroke risk in HCM, did not independently predict death in the HCM group. Instead, older age, heart failure, peripheral vascular disease, renal failure, and coagulopathy were tied to mortality. For clinicians, this means HCM itself should be viewed as a high-risk marker in stroke patients. in addition to the presenting rhythm, including atrial fibrillation, coexisting conditions deserve close attention during the admission.

## Introduction

Hypertrophic cardiomyopathy (HCM) is the most common genetic cardiomyopathy, predominantly affecting the left ventricle (LV) by causing hypertrophy without the presence of other conditions that increase afterload.^1^ Acute ischemic strokes (AISs) are severe thromboembolic complications frequently observed in patients with HCM.^2–4^ The incidence of stroke and other systemic embolic events in this patient population ranges from 0.8% to 1% per year. The heightened risk of AIS in HCM patients is primarily attributed to the presence of atrial fibrillation (AF).^5,6^ However, there are very limited data on the short-term hospital outcomes for patients hospitalized with AIS who have concurrent HCM. Our aim was to evaluate the impact of HCM on the short-term prognosis of patients admitted with AIS and to identify predictors that may contribute to increased all-cause in-hospital mortality.

## Methods

### Data source

We identified hospitalizations with admitting diagnoses of AIS using the Healthcare Cost and Utilization Project’s (HCUP)^7^ National Inpatient Sample (NIS) database between January 1, 2016, and December 31, 2019. The NIS database is one of the largest publicly available all-payer inpatient health care databases in the United States (US). It contains information on >7 million hospital stays each year from over 1000 hospitals nationwide. This de-identified database allows for the querying of individual hospitalizations with primary and multiple secondary discharge diagnoses using the International Classification of Diseases (ICD) codes. It provides a comprehensive resource for analyzing trends in hospitalization, health care costs, quality of care, health care services usage, and outcomes. Institutional review board approval and informed consent were not required for this study, as data were de-identified.

### Study population

We retrospectively identified patients aged ≥18 years hospitalized with a diagnosis of AIS using the ICD, Tenth Revision, Clinical Modification (ICD-10-CM) codes. Hospitalizations with missing data on age, sex, and mortality were excluded. The study population was then divided into two groups: those with a diagnosis of HCM (case group) and those without a diagnosis of HCM (control group). Hospitalizations with an HCM diagnosis were identified using specific ICD-10-CM codes, as detailed in **Supplementary Table 1**. **Figure 1** provides an overview of the study population selection.

**Supplementary Table 1:** International Classification of Diseases, Tenth Revision, Codes for Diagnosis

Diagnosis	Code(s)
Atrial fibrillation	I480, I481, I482, I4891, I4819, I4820, I4821, I4811
Hypertrophic cardiomyopathy	I421, I422
Ischemic stroke	I6300, I63011, I63012, I63013, I63019, I6302, I63031, I63032, I63033, I63039, I6309, I6310, I63111, I63112, I63113, I63119, I6312, I63131, I63132, I63133, I63139, I6319, I6320, I63211, I63212, I63213, I63219, I6322, I63231, I63232, I63233, I63239, I6329, I6330, I63311, I63312, I63313, I63319, I63321, I63322, I63323, I63329, I63331, I63332, I63333, I63339, I63341, I63342, I63343, I63349, I6339, I6340, I63411, I63412, I63413, I63419, I63421, I63422, I63423, I63429, I63431, I63432, I63433, I63439, I63441, I63442, I63443, I63449, I6349, I6350, I63511, I63512, I63513, I63519, I63521, I63522, I63523, I63529, I63531, I63532, I63533, I63539, I63541, I63542, I63543, I63549, I6359, I636, I6381, I6389, I639
Prior stroke	I6930, I69310, I69311, I69312, I69313, I69314, I69315, I69318, I69319, Z8673
Long-term anticoagulant use	Z7901
Coronary artery disease	I2510, I25110, I25111, I25118, I25119

**Figure 1:**
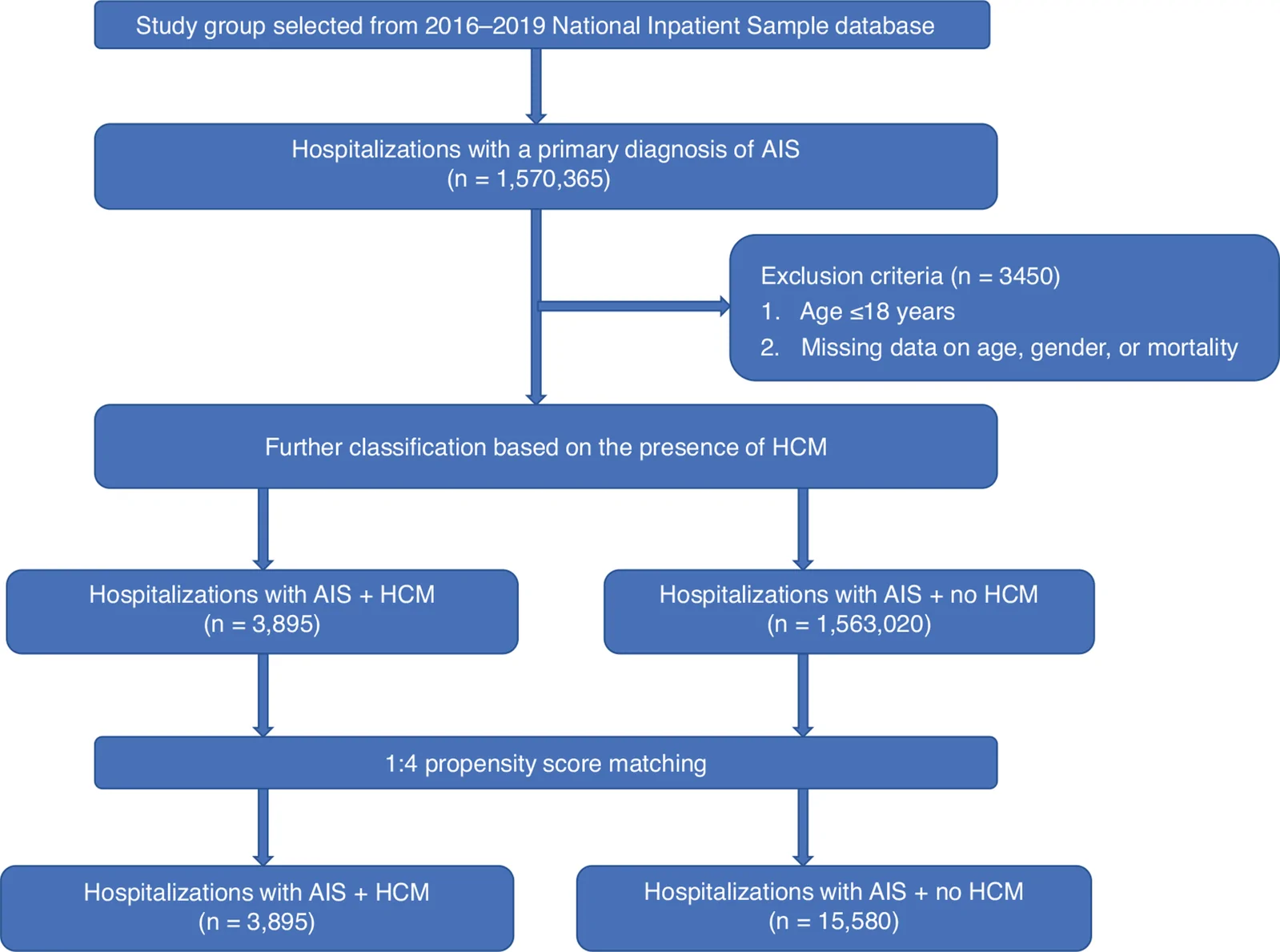
Study population selection algorithm. The study population was selected from the National Inpatient Sample database 2016–2019 using International Classification of Diseases, Tenth Revision, codes, focusing on those who had a primary diagnosis of acute ischemic stroke. After applying the exclusion criteria, 3450 hospitalizations were excluded, and 1,566,915 were included for the final analysis. They were further classified into two groups based on the presence or absence of hypertrophic cardiomyopathy. A 1:4 propensity score match was performed to balance and compare the two groups. *Abbreviation:* AIS, acute ischemic stroke; HCM, hypertrophic cardiomyopathy.

### Statistical analyses

The analysis used weighted variables to account for the NIS database’s sampling design, representing the data of the wider population. Continuous variables were expressed as means (± standard deviations) or medians (interquartile ranges), and categorical variables were expressed as frequencies (percentages). We compared categorical variables in baseline characteristics using the Pearson chi-squared test, while continuous variables were analyzed using Student’s *t* test. *P* < .05 was considered statistically significant. We employed propensity score matching (PSM) to balance baseline characteristics between the two groups. These characteristics included age, sex, race, prior strokes, long-term (current) anticoagulant use, hypertension, diabetes mellitus, AF, congestive heart failure (CHF), coronary artery disease, valvular heart disease, respiratory failure, and coagulopathy. PSM was performed using a 1:4 matching scheme without replacement, employing nearest number matching within a caliper width of 0.2. An absolute standardized difference of <10% (0.1) indicated an adequate balance between the two groups. Multivariate logistic regression analysis was used to assess the association between HCM and hospital outcomes, which included in-hospital mortality and length of hospital stay. All statistical analyses were performed using SPSS version 28 (IBM Corp., Armonk, NY, USA).

## Results

During the study period, 1,566,915 hospitalizations with an index diagnosis of AIS were identified, of which 3895 (0.25%) had a concurrent diagnosis of HCM. Patients in the HCM group were younger (69.9 ± 14.3 vs. 70.2 ± 13.9 years; *P* < .001) and more likely to be female (57.8% vs. 50.1%; *P* < .001) compared to patients without HCM. Patients with HCM had higher rates of AF (odds ratio [OR], 2.3; 95% confidence interval [CI], 2.2–2.5; *P* < .001), valvular heart disease (OR, 2.9; 95% CI, 2.7–3.2; *P* < .001), CHF (OR, 2.2; 95% CI, 2.1–2.4; *P* < .001), and long-term (current) anticoagulation use (OR, 1.4; 95% CI, 1.3–1.6; *P* < .001) compared to those without HCM. Other baseline characteristics are shown in **Table 1**.

**Table 1:** Comparison of Baseline Characteristics of Acute Ischemic Stroke Hospitalizations in Hypertrophic Cardiomyopathy (HCM) and Non-HCM Groups

Variable	HCM, n = 3895 (%)	Non-HCM, n = 1,563,020 (%)	*P* Value
Age (years), mean ± SD	69.9 ± 14.3	70.2 ± 13.9	<.001
Female sex	57.8	50.1	<.001
Congestive heart failure	32.6	18	<.001
Atrial fibrillation	43.8	25.1	<.001
Coagulopathy	6	5	.002
Prior stroke	15.7	15.7	.899
Long-term anticoagulant use	21.2	15.6	<.001
Valvular heart disease	24.5	9.9	<.001
Coronary artery disease	31.2	25.9	<.001
Chronic pulmonary disease	16.9	15.9	.068
Peripheral vascular disease	18.7	9.6	<.001
Hypertension	89.1	84.5	<.001
Diabetes mellitus	33.4	38.8	<.001
Hypothyroidism	15.3	14.4	.102
Renal failure	24.3	17.8	<.001
Liver disease	3.9	1.8	<.001
Hyperlipidemia	62.9	59.7	<.001
Smoking	15.5	18.8	<.001

*Abbreviations:* AIS, acute ischemic stroke; HCM, hypertrophic cardiomyopathy; SD, standard deviation.

After 1:4 propensity matching, the HCM group included 3895 hospitalizations, and the control group included 15,580 hospitalizations. The differences in demographic variables and comorbidities between patients with and without HCM were well balanced, with the absolute mean standardized difference consistently being <10%, as summarized in **Table 2**.

**Table 2:** Baseline Characteristics of Acute Ischemic Stroke Hospitalizations After Propensity Score Matching

Variable	HCM, n = 3895 (%)	Non-HCM, n = 15,580 (%)	Absolute Mean Standardized Difference (%)
Age (years), mean ± SD	69.9 ± 14.3	70.7 ± 14.4	4.8
Female sex	57.8	57.8	0.06
Congestive heart failure	32.6	33.2	1.3
Valvular heart disease	24.5	24.8	0.7
Peripheral vascular disease	18.7	18.2	1.3
Chronic pulmonary disease	16.9	15.9	2.6
Hypothyroidism	15.3	16.5	3.5
Renal failure	24.3	27	6.4
Liver disease	3.9	3.0	4.7
Coagulopathy	6.0	4.7	5.4
Hypertension	89.1	91.4	7.4
Diabetes mellitus	33.4	35.6	4.6
Prior stroke	15.7	14.9	2.2
Long-term anticoagulant use	21.2	21.5	0.9
Coronary artery disease	31.2	33.2	4.4
Atrial fibrillation	43.8	44.5	1.5
Hyperlipidemia	62.9	64.7	3.7
Smoking	15.5	13.9	4.5

*Abbreviations:* AIS, acute ischemic stroke; HCM, hypertrophic cardiomyopathy; SD, standard deviation.

Multivariate logistic regression showed that, among patients hospitalized with AIS, concurrent HCM was significantly associated with higher all-cause in-hospital mortality (adjusted OR [aOR], 1.81; 95% CI, 1.53–2.13; *P* < .001) and longer hospital stays (mean, 6 vs. 5.1 days; *P* < .001) compared to those without HCM **(Table 3)**. Other variables independently associated with mortality in patients hospitalized with AIS include age (aOR, 1.02; 95% CI, 1.01–1.03; *P* < .001), female sex (aOR, 1.27; 95% CI, 1.08–1.50; *P* = .003), CHF (aOR, 1.67; 95% CI, 1.42–1.97; *P* < .001), AF (aOR, 1.66; 95% CI, 1.40–1.97; *P* < .001), coronary artery disease (aOR, 1.23; 95% CI, 1.05–1.46; *P* = .01), renal failure (aOR, 2.45; 95% CI, 2.07–2.90; *P* < .001), liver disease (aOR, 1.98; 95% CI, 1.45–2.71; *P* < .001), and coagulopathy (aOR, 2.39; 95% CI, 1.88–3.03; *P* < .001) **(Table 4)**. In patients with HCM, age (aOR, 1.03; 95% CI, 1.01–1.04; *P* < .001), female sex (aOR, 1.45; 95% CI, 1.06–1.98; *P* = .003), CHF (aOR, 2.07; 95% CI, 1.53–2.79; *P* < .001), peripheral vascular disease (aOR, 1.68; 95% CI, 1.20–2.36; *P* = .002), renal failure (aOR, 1.45; 95% CI, 1.26–2.42; *P* < .001), and coagulopathy (aOR, 3.14; 95% CI, 2.04–4.83; *P* < .001) were found to be predictors of mortality **(Table 5)**. Long-term (current) anticoagulant use (aOR, 0.63; 95% CI, 0.42–0.95; *P* = .03) was found to be protective of all-cause mortality. However, AF (aOR, 0.98; 95% CI, 0.72–1.32; *P* = .89) and prior strokes (aOR, 0.63; 95% CI, 0.38–1.03; *P* = .06) were not found to be independent predictors of mortality.

**Table 3:** In-hospital Outcomes of Acute Ischemic Stroke Hospitalizations with and Without Hypertrophic Cardiomyopathy After 1:4 Propensity Score Matching

Outcome	HCM, n = 3895 (%)	Non-HCM, n = 15,580 (%)
Died	5.9	3.4
Length of stay (days), mean	6.0	5.1

*Abbreviations:* AIS, acute ischemic stroke; HCM, hypertrophic cardiomyopathy.

**Table 4:** Predictors of Mortality in Hospitalizations with Acute Ischemic Stroke After Propensity Score Matching

Variable	aOR (95% CI)	*P* Value
Age	1.02 (1.01–1.03)	<.001
Female sex	1.27 (1.08–1.50)	.003
HCM	1.81 (1.53–2.13)	<.001
Congestive heart failure	1.67 (1.42–1.97)	<.001
Atrial fibrillation	1.66 (1.40–1.97)	<.001
CAD	1.23 (1.05–1.46)	.01
Renal failure	2.45 (2.07–2.90)	<.001
Liver disease	1.98 (1.45–2.71)	<.001
Coagulopathy	2.39 (1.88–3.03)	<.001

*Abbreviations:* AIS, acute ischemic stroke; aOR, adjusted odds ratio; CAD, coronary artery disease; CI, confidence interval; HCM, hypertrophic cardiomyopathy.

**Table 5:** Predictors of Mortality in AIS with Concurrent Hypertrophic Cardiomyopathy Diagnosis

Variable	OR (95% CI)	*P* Value
Age	1.03 (1.01–1.04)	<.001
Female sex	1.45 (1.06–1.98)	.01
Congestive heart failure	2.07 (1.53–2.79)	<.001
Peripheral vascular disease	1.68 (1.20–2.36)	.002
Renal failure	1.75 (1.26–2.42)	<.001
Coagulopathy	3.14 (2.04–4.83)	<.001

*Abbreviations:* AIS, acute ischemic stroke; CI, confidence interval; HCM, hypertrophic cardiomyopathy; OR, odds ratio.

## Discussion

In our current multicenter retrospective study, we found that, among patients admitted with a primary diagnosis of AIS, HCM was associated with increased inpatient mortality and longer hospital stays compared to patients without HCM. Our analysis also identified age, CHF, peripheral vascular disease, renal failure, and coagulopathy as independent predictors of mortality, while AF, long-term anticoagulant use, and prior strokes were not identified as significant independent predictors of mortality.

Stroke remains one of the leading causes of death in the US, with ischemic strokes accounting for nearly 87% of all cases.^8^ Notably, patients with HCM face a high risk of AIS and elevated cardiovascular death rates.^4^ In patients with HCM, the prevalence of AF is as high as 20%,^9,10^ and AF is also associated with increased mortality in this group.^11^ Although guidelines recommend to effectively initiate anticoagulants in HCM patients with documented AF irrespective of any known risk factors to prevent thromboembolic events,^2^ AF occurs in only 20% of these patients.^12,13^ The remaining 80% of patients are still at a higher risk of thromboembolic events. Our study contributes to this knowledge by highlighting that AF was not an independent predictor of mortality in our study group when compared to the propensity-matched control group.

Prior studies have shown that HCM patients surviving into their seventh decade are at a low risk for disease-related mortality, including sudden death.^14,15^ We believe this is related to the fact that higher-risk patients might have died earlier in life or because sudden deaths might have been prevented by prophylactically implanted intracardiac defibrillators.^16^ However, advanced age increases the risk of cardiovascular or non-cardiovascular complications such as hypertension, hyperlipidemia, diabetes, peripheral vascular diseases, coronary artery disease, and AF, all of which can contribute to increased mortality in AIS patients with HCM.^9,17^ This is supported by our study finding that age and peripheral vascular disease are predictors of mortality in patients hospitalized with AIS who had HCM.

Our study found that the in-hospital mortality rate after AIS was 5.9% in patients with HCM, which may underestimate the overall impact of HCM on AIS survival, given that most stroke-related deaths (64%) occur outside acute care hospitals.^8^ In a minority of patients with HCM, severe microvascular dysfunction leads to adverse LV remodeling and eventually systolic dysfunction.^18^ HCM with LV systolic dysfunction is associated with a significantly increased risk of sudden cardiac death.^19,20^ This finding is supported by our observation that AIS patients with HCM had higher baseline rates of CHF compared to patients without HCM, and CHF was a predictor of all-cause mortality. Although the 2024 American Heart Association/American College of Cardiology HCM guidelines recommend prophylactic implantable cardioverter-defibrillator implantation in HCM patients with LV systolic dysfunction,^2^ we believe that early use of cardiac resynchronization therapy can be considered in these patients to reduce overall all-cause and cardiovascular mortality.

The presence of AF in HCM patients is poorly tolerated. The increased LV filling pressures can be transmitted to the left atrium (LA) through mitral regurgitation, leading to atrial myopathy.^21^ LA myopathy can lead to thrombus formation, but thrombi can also form in sinus rhythm through this intrinsic pathway.^22,23^ The increased risk of thrombus formation in patients already admitted with AIS can multiply the risk of thromboembolism. A recent observational study showed that AF was significantly associated with a higher risk of in-hospital mortality in patients admitted with AIS, whereas long-term (current) use of anticoagulants was associated with a reduced risk of death.^24^ Our analysis also found AF to be associated with in-hospital mortality in patients admitted with AIS. However, in patients who have concurrent HCM, AF was not independently associated with in-hospital mortality. From our analysis, we can safely interpret that HCM patients admitted with AIS are at a higher risk of mortality, with or without the presence of AF.

The LV outflow tract obstruction and systolic anterior motion in HCM^25^ can result in pressure transmission from the LV side to the right side, ultimately leading to pulmonary hypertension (PH). The presence of PH in HCM patients has been associated with elevated morbidity and mortality in hospitalizations with heart failure.^26,27^ We hypothesize that the presence of elevated risk factors, such as those mentioned previously, in patients hospitalized for AIS is associated with longer hospital stays in HCM patients.

### Limitations

This study, based on the NIS database, has inherent limitations, including the absence of granular data on individual patients with HCM. Although PSM was applied, pertinent clinical factors such as stroke severity (National Institutes of Health Stroke Severity), HCM phenotype (obstructive vs. non-obstructive), LV ejection fraction, adherence to anticoagulant use, and imaging data could not be accurately obtained from the NIS database, which might have influenced our study outcomes. Given AF’s established relationship with stroke outcomes, potential explanations such as the effect of anticoagulation, coding limitations, or survival bias, and with the aforementioned limitations of NIS, our study results should be interpreted cautiously. Second, reperfusion therapies, specifically intravenous thrombolysis (tissue plasminogen activator) and mechanical thrombectomy, are major determinants of outcomes in AIS and represent potential confounders in our analysis. Third, we did not formally test an HCM and AF interaction term in a unified multivariable model. The differential association of AF with mortality in the overall propensity-matched cohort versus the HCM-only subgroup may represent true effect modification, but this should be interpreted cautiously, given the limitations of the NIS database and the relatively small sample size of the HCM subgroup. The rarity of HCM compared to the general population might have influenced the finding that AF is not a predictor of mortality in AIS patients with HCM in our study. Lastly, our study may underestimate the overall mortality rates of AIS patients with HCM, as it only included hospitalizations with AIS as the primary diagnosis code.

## Conclusion

AIS patients with concurrent HCM are at a higher risk of increased in-hospital mortality and longer hospital stays when compared to a propensity score–matched control population without HCM. In patients with HCM, age, CHF, peripheral vascular disease, renal failure, and coagulopathy were found to be predictors of mortality, whereas AF was not a predictor of mortality. Further prospective studies are required to confirm our findings and establish effective management strategies to reduce mortality in AIS patients with HCM, regardless of documented AF.
